# The potential of plants as a system for the development and production of human biologics

**DOI:** 10.12688/f1000research.8010.1

**Published:** 2016-05-19

**Authors:** Qiang Chen, Keith R. Davis

**Affiliations:** 1 Biodesign Institute and School of Life Sciences, Arizona State University, Tempe, AZ, USA; 2 The Johnson Center for Innovation and Translational Research, Indiana University, Bloomington, IN, USA

**Keywords:** plant-made biologics, plant-host engineering, plant expression platforms

## Abstract

The growing promise of plant-made biologics is highlighted by the success story of ZMapp™ as a potentially life-saving drug during the Ebola outbreak of 2014-2016. Current plant expression platforms offer features beyond the traditional advantages of low cost, high scalability, increased safety, and eukaryotic protein modification. Novel transient expression vectors have been developed that allow the production of vaccines and therapeutics at unprecedented speed to control potential pandemics or bioterrorism attacks. Plant-host engineering provides a method for producing proteins with unique and uniform mammalian post-translational modifications, providing opportunities to develop biologics with increased efficacy relative to their mammalian cell-produced counterparts. Recent demonstrations that plant-made proteins can function as biocontrol agents of foodborne pathogens further exemplify the potential utility of plant-based protein production. However, resolving the technical and regulatory challenges of commercial-scale production, garnering acceptance from large pharmaceutical companies, and obtaining U.S. Food and Drug Administration approval for several major classes of biologics are essential steps to fulfilling the untapped potential of this technology.

## Introduction

**Figure 1.**
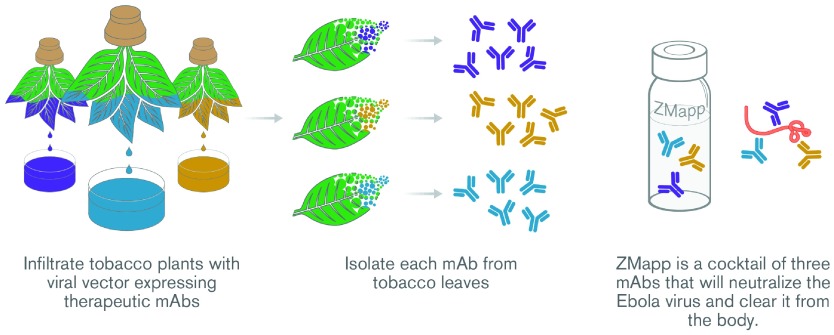
Overview of the production of ZMapp™ in *Nicotiana benthamiana* plants.

A significant chapter in the 2014–2016 Ebola outbreak came from the survival of two infected American health aid workers. Dr Kent Brantly and Nancy Writebol’s Ebola infection quickly escalated to the point where they thought they were dying. Remarkably, their condition dramatically improved soon after receiving an experimental drug called ZMapp™ ^1^. ZMapp™ is a cocktail of three monoclonal antibodies (mAbs) produced in plants ( Figure 1). The fact that plant-produced proteins can be life-saving drugs has brought renewed attention to the field of plant-made biologics (PMBs). Why are plants used to produce ZMapp™ and other biologics? Will plant-based expression systems outcompete mammalian cell culture systems as a general platform for biologics production in the future? What are the remaining challenges that have to be overcome for this technology to fulfill its potential? These are some of the questions that have been repeatedly asked by the general public and drug development strategists. In this review, we aim to address these questions.

## Why use plants?

Protein-based biologics comprise the largest and fastest growing class of pharmaceutical products. Currently, the majority of human biologics are produced in mammalian and microbial cell cultures. Biologics produced in cell cultures require capital-intensive facilities, fermenters, expensive downstream processing, cold storage and transportation, and sterile delivery methods. These limitations encourage the development of alternative production systems.

In contrast to animal- and microbial-based cell culture platforms, early studies of PMBs emphasized the advantages of plants with respect to their low production costs, high scalability in upstream protein expression, and increased safety ^2^. Given that plants rarely carry human or animal pathogens, the risk of introducing pathogens is far lower compared to mammalian cell production. Another advantage of biologic production in plants is that it does not require capital-prohibitive facilities, bioreactors, and expensive culture media but can be easily scaled in relatively inexpensive greenhouses with simple mineral solutions. Thus, lower manufacturing costs have been widely assumed as an innate advantage of plant-based production platforms.

It is crucial to understand the true cost of PMBs because manufacturing cost does have an impact on the market acceptability and profitability of a product. This has been a controversial topic, as information on the actual costs of producing PMBs at industrial scale has not been readily available. Recent case studies by Tusé *et al.* provide urgently needed economic evaluations of the current PMB platforms. Their studies revealed that plant-based platforms can substantially reduce the production cost of biologics compared with traditional platforms, with upstream costs of goods as low as $1.00–2.00 per kilogram of protein ^3^. However, it is important to note that the cost of downstream processing for PMBs, especially for parenteral applications, is estimated to be similar to that of other production systems. These studies provide the first direct evidence to support the long-held belief that plants can lower the cost of biologic production.

New plant expression systems also offer the flexibility and speed that cannot be matched by production technologies based on mammalian cell culture. This is due to innovations in expression-vector development, particularly vectors for transient expression. The development of ‘deconstructed’ viral vector systems (e.g. magnICON, geminiviral, and pEAQ) has successfully addressed the challenges of insufficient protein expression levels, consistency, and speed of biologic production in plants ^4– 6^. For example, transient expression with deconstructed viral vectors allows the production of up to 5 milligrams of mAb per gram of fresh leaf weight within 2 weeks, in contrast to using a process that requires generating and selecting transgenic plants; this can take from months to years. Moreover, transgenic plant production often results in low and inconsistent protein yield ^7^. The rapid and high-level protein production capability of transient expression technology makes it the optimal system to produce milligram and gram levels of biologics for pre-clinical studies. ‘Bridge’ versions of these vectors have also been developed for scale-up manufacturing of biologics in stable transgenic plants ^8, 9^. Thus, ‘deconstructed’ viral vectors offer a set of versatile tools that can rapidly evaluate biologic candidates and then transition them to large-scale commercial manufacturing.

Host engineering is another source of innovation that equips plant-expression systems with additional advantages. For example, the minor differences in N-glycosylation between plant and human cells have been a major concern, as they may trigger the production of plant-glycan-specific antibodies that could reduce therapeutic efficacy or cause adverse effects. By knocking out specific genes required for plant-specific glycosylation patterns and inserting mammalian glycosylation genes, glycoengineering generates plant hosts that produce mAbs with authentic human N-glycans ^10^. Furthermore, plant-produced mAbs also have a degree of glycan homogeneity that cannot be produced by mammalian cells or by *in vitro* treatments ^11, 12^. This represents an advantage during the regulatory approval stage of product development. The availability of a portfolio of plant lines that can produce biologics with tailor-made mammalian N-glycans on demand provides the opportunity to develop vaccines and therapeutics with more potent efficacy or safety than those produced in other production platforms.

Plant cells may also provide a novel vehicle for oral delivery of biologics. Conventional biologics are produced by a costly downstream process and require continuous refrigeration, referred to as the ‘cold-chain’, for their transport and storage plus sterile needles for injection. Oral delivery of protein drugs is appealing but has been elusive due to their denaturation and degradation in the digestive system and inability to cross the gut epithelium and subsequently deliver to target cells. Since human digestive enzymes cannot hydrolyze the glycosidic bonds in the carbohydrates of the plant cell wall, plant cells can protect expressed biologic proteins from acids and enzymes in the stomach by bioencapsulation, allowing them to enter the gut lumen where they are enzymatically released by gut commensal bacteria ^13^. Recent studies also demonstrated that orally delivered plant cell-encapsulated protein drugs can cross the gut epithelium to enter the bloodstream. Depending on the specific targeting sequences they are fused to, the orally delivered protein drugs can either induce tolerance against inhibitory antibody production associated with their injection or enter the circulatory system to treat diabetes, hypertension, or other metabolic diseases ^13– 15^. Encapsulated protein drugs in plant cells have been found to maintain their pharmacological efficacy several years after storage at room temperature ^16^.

These striking results suggest that plant-cell-encapsulated biologics may represent an ambient temperature-stable product that can be ingested by patients. These temperature-stable products allow the practical implementation of healthcare programs in regions where the ‘cold-chain’ and other logistical challenges limit the delivery of medical supplies. The commercial implementation of this strategy in the developed world would also reduce the cost associated with downstream processing, cold storage, and transportation. However, the regulatory challenges of this technology must be addressed, as vaccines or therapeutic drugs are required to have strictly controlled dosages, which is currently difficult to achieve with cell-encapsulation technology. Nevertheless, as new expression vectors provide more consistent biologic accumulation per unit of plant mass, this strategy may eventually offer an attractive future option for biologic delivery in both the developed and the developing world.

## Successes and remaining challenges

The story of ZMapp™ highlights how innovations in several basic technologies can come together and lead to the development of a life-saving drug candidate ^2^. The development of magnICON vectors allowed for the rapid and high-level accumulation of anti-Ebola GP1 mAbs in *Nicotiana benthamiana* plants ^17, 18^. Host optimization permitted the production of these mAbs with various mammalian glycoforms, leading to the discovery that plant-derived anti-Ebola mAbs with homogenous GnGn mammalian glycans have a superior potency to their mammalian-produced counterparts ^12, 19^. Progress in downstream processing of plant materials allowed for the effective extraction and purification of these mAbs ^20, 21^. Efficacy studies in rhesus macaques demonstrated that a three-mAb cocktail was able to rescue 100% of animals even when given 5 days after a lethal Ebola challenge ^22^. All of these paved the way for the formulation of ZMapp™ and its compassionate use in Brantly, Writebol, and five other human patients during the Ebola outbreak. A clinical trial has recently been concluded in the U.S., Liberia, Sierra Leone, and Guinea for ZMapp™ by the U.S. National Institutes of Health to assess the safety and efficacy of ZMapp™.

Another promising application of plant-based expression of biologics is the development of influenza virus-like particle (VLP)-based vaccines. Studies in this area have demonstrated the superiority of plant systems over other manufacturing platforms in their simplicity and speed for controlling potential pandemics ^23^. An effective pandemic influenza vaccine needs to be produced in the shortest achievable timeframe to halt the spread of the new strain. VLPs comprising hemagglutinin (HA) alone are the simplest candidates for influenza pandemic vaccines because they require only the HA coding sequence of the pandemic strain for expression, impose fewer constraints on process and product characterization, and lower the risk of failure when production processes need to be adapted for a new viral strain ^23^. However, producing VLPs based on HA alone is not feasible in animal cells because HA binds to the sialylated glycoproteins on the cell ^24^. Plant cells provide a unique advantage for producing this VLP type because plant glycoproteins are not sialylated. The use of plants also avoids the supply issue of eggs in the event of massive culling of chickens, or if the influenza virus is lethal to embryonated eggs. Most importantly, the need for strain adaptation is eliminated when using plants, shortening the time required for vaccine production. In a real-life test in response to an unexpected outbreak of a novel A/H1N1 influenza virus, it took only 2 weeks to obtain infiltrated plants that expressed high levels of HA of the new strain and another 5 days to obtain the first purified lot of the vaccine from the date that the HA sequence of this strain became available ^23^. This is in stark contrast with all current manufacturing technologies, which rely on strain adaptation, a process that requires an additional 4–6 months before vaccine production can be initiated. Efficacy studies in mice indicate the plant-derived VLPs have equivalent if not superior potency compared to vaccines produced in eggs ^23^. The plant-derived vaccine candidates against various influenza strains (e.g. H5N and H1N1) have been tested in phase I and phase II human clinical trials. They were found to be safe and well tolerated, and the potency was among the most effective of the industry ( www.medicago.com). This indicates that plant-based platforms provide an ideal system to produce biologics in response to emerging or re-emerging pathogens with unpredictable and frequent genetic drift or stockpile for bioterrorism threats.

The advantages of plant-based systems have been further demonstrated in the case of glucocerebrosidase (GCD), a therapeutic enzyme for treating Gaucher’s disease. Mammalian cell-produced GCD requires *in vitro* N-glycan processing to achieve the desired efficacy, substantially complicating the manufacturing process and increasing the production cost. In contrast, the plant-produced GCD already contains the required glycoform, eliminating the costly N-glycan processing and possibly resulting in better and more consistent efficacy ^25^. As a result, the U.S. Food and Drug Administration (FDA) and other regulatory agencies have approved the use of plant-produced GCD (commercially named ELELYSO™) to treat Gaucher’s patients ^26^. This is the very first PMB therapeutic ever approved by the FDA and it has been marketed in the U.S., Canada, and many Latin-American countries. A new oral-delivered version of GCD has been tested in animal models ^27^ and in a phase I and a phase II human clinical trial ( www.protalix.com). Results showed that the levels of GCD in the blood circulation of Gaucher’s patients were similar to those of healthy individuals when a juice containing lyophilized, GCD-containing carrot cells was consumed daily. The needle-free delivery of GCD will improve patients’ quality of life, encourage treatment compliance, and allow the implementation of therapy in areas where medical supplies are limited.

The application of PMBs has reached beyond the traditional realm of vaccines and therapeutic proteins. For example, plant-made bacterial colicins were recently shown to be very effective as food additives for controlling pathogenic bacteria in food products ^28^. Even applied at low concentrations, the plant-derived colicins were shown to be highly and broadly active against all major pathogenic *Escherichia coli* strains that cause food poisoning. The production cost was estimated to be $1.00 per gram of purified colicins, indicating its commercial viability. The FDA’s “no questions” response letter to the commercialization request of plant-made colicins signals its potential regulatory approval. The commercialization of this product may significantly reduce bacterial enteric infections worldwide, as, currently, no effective methods are available to control pathogenic bacteria in the food chain.

There are still technical and regulatory challenges that must be overcome to fulfill the potential of PMBs. For example, the current manufacturing capacity of PMBs is still limited. This limitation was revealed when the demand for ZMapp™ during the 2014–2016 Ebola outbreak couldn’t be met even though a commercial-scale PMB facility devoted its full capacity to producing ZMapp™. Despite the successes of large-scale production of several PMBs, large-scale downstream processing of PMBs from whole plants remains challenging. Plants typically produce more solid debris than other organisms, and some plant species, such as those in the tobacco family, contain high levels of phenolics and alkaloids. As a result, clarification of plant extracts often cannot be achieved simply by a single round of filtration as is the case for mammalian cells, and direct loading of plant extracts onto chromatography resins may cause resin fouling ^20^. As such, several rounds of filtration with multiple types of filters are required to remove plant particulates and/or plant compounds ^29^. While these technologies can be scaled up to a certain extent, further optimization or the introduction of new processing methods is required since the full potential of PMB production on an agricultural scale demands a processing platform with extraordinarily large-scale capabilities.

Since the launch of the PMB field in the early 1990s, there has been significant skepticism that a PMB product was ever going to be developed; this has now been done. The lingering criticism of the PMB field is the lack of approved human products in major biologic categories after more than 25 years of active research and development. To date, ELELYSO™ is the only PMB that has been approved by the FDA, and outside the compassionate use of ZMapp™ in human patients, no plant-made mAbs or vaccines have yet been licensed as pharmaceutical products for human use, albeit a plant-made mAb (CaroRx) that prevents adhesion of decay-causing bacteria to the tooth surface was approved as a medical device. The previous lack of a clear approval pathway is partially responsible, as the novelty and complexity of this technology caused uncertainty and confusion on how PMBs would fit into the regulatory agencies’ structured framework for biologics. The approval of ELELYSO™ by the FDA has paved a clear regulatory pathway specific for PMBs, especially for those derived from cultured plant cells, and should also streamline the approval of several whole-plant-made mAbs and vaccines that have shown safety and efficacy in human clinical trials ^30^. The past uncertain regulatory environment also contributed to the decision by large pharmaceutical companies to forego PMBs. The recent progress made in the PMB field has, however, slowly warmed up their interests in the PMBs themselves or their production technologies. For example, Pfizer entered into an agreement to license the worldwide rights for commercializing ELELYSO™. Other pharmaceutical companies have also begun to show interest in PMBs through buyouts and partnerships ^31^. The encouraging story of ZMapp™ has also sparked new interests and promoted several large government investments to expand the capacity of producing biologics from plants under current Good Manufacturing Practice (cGMP) regulations ^30, 32^.

## Conclusions

Plant production systems not only offer the traditional advantages of proper eukaryotic protein modification, low costs, high scalability, and increased safety but also allow the production of biologics at unprecedented speed to control potential pandemics or with specific post-translational modifications for superior potency or safety. These advantages make plants a superior alternative production system for biologic production. However, it is unlikely that plants will replace mammalian cells as the primary host for biologic production in the foreseeable future. Instead, plant-based systems will likely have a broad range of special niches for the production of specific biologics. Plants would be a system of choice to produce biosimilars because of their large capacity to rapidly generate biologics at low cost, the ability to easily incorporate post-translational modifications, and their low contamination risks with animal or human pathogens. For similar reasons, plants also offer an optimal system to produce biologics requiring extraordinarily large-quantity production that have relatively low profit margins. Plants will also be essential for producing safer and more effective biobetters due to the flexibility of producing biologics with specific and homogeneous mammalian glycoforms that cannot currently be easily produced by other cell culture systems. For the ultimate adoption and success of PMB technologies, it is crucial to overcome the scalability issue in downstream processing and vastly expand the approval pipeline of plant-made proteins in several key classes of biologics, such as mAbs and vaccines, within the next decade. Overall, the favorable outcome with ZMapp™ and the involvement of big pharma are promising signs and a harbinger of new optimism for the PMB field.

## Abbreviations

PMBs, plant-made biologics; mAb, monoclonal antibody; VLP, virus-like particle; HA, hemagglutinin; GCD, glucocerebrosidase; FDA, US Food and Drug Administration; cGMP, good manufacturing practice.
